# Impact of Bone Marrow miR-21 Expression on Acute Myeloid Leukemia T Lymphocyte Fragility and Dysfunction

**DOI:** 10.3390/cells9092053

**Published:** 2020-09-08

**Authors:** Douâa Moussa Agha, Redouane Rouas, Mehdi Najar, Fatima Bouhtit, Hussein Fayyad-Kazan, Laurence Lagneaux, Dominique Bron, Nathalie Meuleman, Philippe Lewalle, Makram Merimi

**Affiliations:** 1 Laboratory of Experimental Hematology, Jules Bordet Institute, Université Libre de Bruxelles, 1000 Bruxelles, Belgium; douaa.moussa@gmail.com (D.M.A.); redouane.rouas@bordet.be (R.R.); bouhtitfatima@gmail.com (F.B.); dominique.bron@bordet.be (D.B.); nathalie.meuleman@bordet.be (N.M.); philippe.lewalle@bordet.be (P.L.); 2 Osteoarthritis Research Unit, University of Montreal Hospital Research Center (CRCHUM) and Department of Medicine, University of Montreal, Montreal, QC H2X 0A9, Canada; mnajar@ulb.ac.be; 3 Genetics and Immune Cell Therapy Unit, Faculty of Sciences, University Mohammed Premier, Oujda 60000, Morocco; 4 Laboratory of Cancer Biology and Molecular Immunology, Faculty of Sciences I, Lebanese University, Hadath 90656, Lebanon; hfayyadk@gmail.com; 5 Laboratory of Clinical Cell Therapy, Jules Bordet Institute, Université Libre de Bruxelles, 1070 Bruxelles, Belgium; laurence.lagneaux@bordet.be

**Keywords:** AML, T lymphocytes, extracellular vesicles, miR21, apoptosis, immunosuppression

## Abstract

Background: Acute myeloid leukemia (AML) is a hematopoietic malignancy in which antitumor immunity is impaired. The therapeutic management of AML requires understanding the mechanisms involved in the fragility and immune dysfunction of AML T lymphocytes. Methods: In this study, T lymphocytes from healthy donors (HD) and AML patients were used. Extracellular vesicles (EVs) from leukemic cells were screened for their microRNA content and impact on T lymphocytes. Flow cytometry, transcriptomic as well as lentiviral transduction techniques were used to carry out the research. Results: We observed increased cell death of T lymphocytes from AML patients. EVs from leukemia myeloid cell lines harbored several miRNAs, including miR-21, and were able to induce T lymphocyte death. Compared to that in HD, miR-21 was overexpressed in both the bone marrow fluid and infiltrating T lymphocytes of AML patients. MiR-21 induces T lymphocyte cell death by upregulating proapoptotic gene expression. It also increases the immunosuppressive profile of T lymphocytes by upregulating the IL13, IL4, IL10, and FoxP3 genes. Conclusions: Our results demonstrate that miR-21 plays a significant role in AML T lymphocyte dysfunction and apoptosis. Targeting miR-21 may be a novel approach to restore the efficacy of the immune response against AML.

## 1. Introduction

Acute myeloid leukemia (AML) is the most prevalent malignant myeloid tumor, primarily occurring in elderly patients and characterized by proliferation of immature myeloid precursors [1]. Molecular, genetic and cytogenetic abnormalities cause clonal expansion of early hematopoietic progenitor cells and obstruct normal bone marrow (BM) hematopoiesis [2]. There is increasing evidence that the immune microenvironment plays a fundamental role in the outcome of leukemia [1,2]. Tumor-infiltrating T lymphocytes (TILs) are present in many human cancers and play a crucial role in the recognition and elimination of tumor cells [3,4]. A number of immune evasion mechanisms orchestrated by tumors specifically inhibit T cell immunity, such as accumulating T regulatory suppressor cell populations and repolarization of the immune response [5,6,7]. The number of successful immune-based therapies for various solid tumors has surged in the past decade. Several new approaches are being actively pursued to develop immunotherapeutic strategies in AML, including tumor-associated antigen (TAA)-specific T cells, natural killer (NK) cell therapy, vaccines, antibody immunotherapy, checkpoint blockade, T regulatory (Treg) suppression, hypomethylating agents and, more recently, CAR-T cells [8]. Contrary to B cell lymphoid malignancies, the promise of CAR-T cells in AML is hampered by the lack of blast-specific antigens. The targeting of leukemic blasts usually mediates sustained eradication of normal myelopoiesis [9]. Currently, the major challenge remaining in the development of effective immunotherapies in AML is overcoming the heterogeneity of the disease and the many immunosuppressive mechanisms exerted by AML blasts.

Within the past few years, numerous studies have demonstrated that the communication between various types of tumor microenvironment (TME) cells and cancer cells is regulated by a unique category of short transcripts that do not encode proteins but do regulate protein expression [10,11]. MicroRNAs (miRNAs) are small endogenous noncoding RNA molecules consisting of 21–25 nucleotides and are known to play a crucial role in cancer and immunity [12,13]. In the context of AML, there is clear evidence that miRNAs play an important role in pathogenesis and drug resistance [14,15,16]. MiR-21 is upregulated in several cancers, including blood cancers such as chronic lymphocytic leukemia, lymphoma, multiple myeloma, and AML [17]. Considered protumoral, miR-21 promotes proliferation in AML by targeting Krüppel-like factor 5 [18]. In addition to its role as an oncogene, miR-21 seems to play an important role in immune cell differentiation and dysfunction in certain cancer types. In our laboratory, using lentiviral transduction of fresh cord blood T cells, we previously demonstrated that miR-21 had a positive effect on FOXP3 expression levels [19] and conferred a regulatory T cell phenotype [20]. Published data revealed that miR-21 was highly expressed on CCR6+ Tregs and important for their dominant enrichment in tumor tissue, which was closely related to the poor prognosis of breast cancer patients. Inhibition of miR-21 significantly reduced the proliferation of CCR6+ Tregs in vitro, altered the enrichment of CCR6+ Tregs in the tumor mass and exerted an effective antitumor effect on CD8+ T lymphocytes [21]. MiR-21 promotes macrophage repolarization from the M1 phenotype to the M2 phenotype [22]. In esophageal squamous cell carcinoma (ESCC), the association between high miR-21 expression and dysregulation of helper T lymphocytes was established with the impairment of antitumor immunity [23]. Our understanding of the immune landscape is an emerging area with great promise for enhancing clinical decisions in AML and developing immunotherapies. In this study, we showed the fragility of lymphocytes from AML patient and attempted to elucidate the link between EV miRNAs, especially miR-21, released by myeloid malignant cells and this phenomenon. Subsequently, we showed that miR-21 is highly expressed in the BM of AML patients and used transduction experiments to show its impact on the fragility and dysfunction of T lymphocytes in AML patients.

## 2. Materials and Methods

### 2.1. Patients

A total of 27 patients newly diagnosed with AML were recruited between 2010 and 2013 at the Jules Bordet Institute (Brussels, Belgium). A total of 11 age- and sex-matched healthy individuals were included as controls. The present study was approved by the Medical Ethics Committee of Jules Bordet Institute (Brussels, Belgium), and every patient provided written informed consent. The characteristics of the 27 AML patients are listed in Table 1.

### 2.2. Bone Marrow and Peripheral Blood T Lymphocyte Isolation

Samples of blood (~15 mL) or BM aspirations (5 mL) were harvested from the patients and mixed with PBS (1:1). The samples were added to gradient centrifugation liquid, Ficoll Paque Plus (Sigma-Aldrich, Overijse, Belgium; Merck Millipore, Overijse, Belgium), and centrifuged at 300× *g* for 20 min at 4 °C. The mononuclear cells (MNCs) were present in a layer between the PBS and Ficoll solution, and this cell layer was harvested. The MNCs were incubated with an anti-human CD3 antibody coated with magnetic beads (human CD3 MicroBeads; Miltenyi Biotec, Leiden, The Netherlands) at 4 °C for 20 min. The T lymphocytes were identified as CD3-positive cells using flow cytometry, and the purity of the cells was ≥95%.

### 2.3. Cell Culture and Cell Death Assay

The isolated T lymphocytes and human leukemia K562, HL60 and KG1 cells (purchased from Sigma) were maintained in medium containing 10% fetal bovine serum (FBS; Thermo Fisher Scientific, Merelbeke, Belgium) and cultured at 37 °C in humidified air containing 5% CO_2_. T lymphocyte cell death was assessed by an annexin-V-FITC/propidium iodide (PI) and annexin-V-APC/7-AAD-based apoptosis detection kit from BD Biosciences according to the manufacturer’s instructions. Cells were seeded in 12-well plates in the presence of 5 μg/mL phytohemagglutinin (PHA-L, Sigma-Aldrich) and 20 U/mL IL-2 (from Sigma). After six days, the cells were harvested, washed twice with PBS-EDTA, stained with annexin-V/PI and analyzed in a FACS machine (NAVIOS-Beckman Coulter, Suarlée, Belgium), and the data generated were analyzed by KALUZA software (Beckman Coulter).

### 2.4. Cell Transduction

Lentiviral vector packaging and transduction were performed as we described previously with slight modifications [24], and the lentiviral vectors were produced by the GIGA viral vector platform from Liege University (Belgium). Human pre-miRNA expression lentivectors (lenti-miRNAs) expressing a control or miR-21 were purchased from System Biosciences (Uden, The Netherlands). After their isolation and activation with PHA and IL2, CD3+ cells were transduced with LV-hsa-miR-21 (multiplicity of infection, MOI = 10) in the presence of polybrene (8 μg/mL, from Sigma). The transduction efficiency was analyzed by cytometry after 48 h, and the percentage of GFP+ cells compared to total cells was calculated. To inhibit miR-21, we used the LentimiRa-Off-hsa-miR-21 vector expressing anti-sense miR-21 (Cat No. mh3032; Applied Biological Materials Inc., Richmond, BC, Canada). The pLenti-III-mir-Off Control Vector was used as a control (Cat No. m007; Applied Biological Materials Inc.). All vectors used in this study also contained a GFP reporter.

### 2.5. Extracellular Vesicle Purification and Analysis

The myeloid leukemia cell lines were cultured in serum-free RPMI-1640 medium and 2% Exo-FBS™ exosome-depleted fetal bovine serum (System Biosciences, Palo Alto, CA, USA) for 48 h, and the cell culture medium (CCM) was collected and centrifuged at 300× *g* for 10 min. EVs were isolated using an exoEasy Maxi Kit (Qiagen, Antwerpen, Belgium). The supernatant was ultracentrifuged using a W32Ti rotor (L-80XP; Beckman Coulter, Brea, CA, USA). PBS was removed, and the EVs were resuspended in 100 µL of PBS. All centrifugation steps were performed at 4 °C. Vesicle suspensions with concentrations between 10^7^/mL and 10^9^/mL were examined using a NanoSight NS300 (NanoSight Ltd., Amesbury, UK) equipped with a 405 nm laser to determine the size and quantity of the isolated particles. A 60-s video was taken with a frame rate of 30 frames/s, and particle movement was analyzed using nanoparticle tracking analysis (NTA) software (version 2.3; NanoSight Ltd.). RNA was extracted from EVs using a Total Exosome RNA and protein isolation kit (Invitrogen, Merelbeke, Belgium; Cat No. 4478545).

### 2.6. Bone Marrow Bodily Fluid Sampling and miRNA Extraction

At presentation, BM aspiration samples were collected in EDTA tubes and processed within 1 h of collection for miRNA detection. BM bodily fluid samples were centrifuged at 1200× *g* for 10 min at 4 °C to pellet the hematopoietic cells; the supernatant was then transferred into microcentrifuge tubes, followed by a second centrifugation at 12,000× *g* for 10 min at 4 °C. The supernatant was transferred to RNase/DNase-free tubes and stored at −80 °C. Total RNA was isolated from plasma samples using a mirVana™ PARIS™ kit (Thermo Fisher Scientific, Merelbeke, Belgium) according to the manufacturer’s instructions. Briefly, total RNA was extracted from 600 μL of human BM plasma. Each sample was eluted in 100 μL of RNase-free water and concentrated to a final volume of 20 μL by using an Eppendorf Concentrator Plus 5301 (Eppendorf, Aarschot Belgium). The RNA sample concentration was quantified by a NanoDrop ND-1000 (Thermo Fisher Scientific, Merelbeke, Belgium).

### 2.7. MicroRNA Expression Profile

A three-step procedure was used in this new study to profile miRNAs in EVs as previously described [19,20,24].

### 2.8. TaqMan miRNA Assay for Individual miRNAs

The expression of miR-21 was determined using the TaqMan miRNA assay as described previously [19,20,24]. The expression levels of miRNAs were calculated using the 2−ΔΔCq method, and miR-48 and miR-425 were used as internal references. The primer sequences were as follows: miR-21 forward, 5′-TAGCTTATCAGACTGATGTTGA-3′ and reverse, 5′-AACGCTTCACGAATTTGCGT-3′. The delta Ct ± SD (standard deviation of the average delta Ct of the group) calculated for the patients was compared with that of the healthy control group and tested for statistical significance.

### 2.9. Transcriptomic Profile Array Analysis

A Human Cell Death Pathway Finder™ RT^2^ Profiler™ PCR Array (88 genes) from Qiagen (Frederick, MD, USA), apoptotic pathways (92 genes), T cell receptor genes (92 genes), Treg pathways (92 genes) and Human Cytokines and Receptors (92 genes) as well as a StellArray Gene Expression System purchased from Lonza (Verviers, Belgium) were used to screen a panel of 366 genes representative of ten different signals of cell death and T cell immune response pathways. Total RNA was isolated from the LV-Ctrl and LV-miR-21 groups using TRIzol reagent (Thermo Fisher Scientific, Waltham, MA, USA) according to the manufacturer’s protocol. RNA was quantified by a NanoDrop, and the quality was assessed by visualizing 18 S and 28 S ribosomal RNA bands separated by 1% agarose with ethidium bromide staining. The first-strand cDNA was mixed with 2×RT^2^ SYBR Green qPCR Master Mix and ddH2O. qPCR was performed on an Applied Biosystems StepOnePlus™ Real-Time PCR System according to the RT2 Profiler PCR Array instructions under the following conditions: 95 °C for 10 min, followed by 40 cycles at 95 °C for 15 s and 60 °C for 1 min. Two housekeeping genes (B2M and 18S) were used for normalization of the sample data. Microarray data were normalized to the housekeeping genes by calculating the ΔCt for each gene of interest in the plate. Fold changes in gene expression, scatterplots and heatmaps were analyzed and generated by using an RT2 PCR array. Genes in miR-21 groups with expression fold changes greater than 1.3 compared with the negative control groups were considered significant.

### 2.10. Reverse Transcription-Quantitative Polymerase Chain Reaction

To validate the expression changes in genes of interest that had fold changes of more than 1.3 in AML T lymphocytes compared to healthy donor (HD) cells, real-time PCR was performed. Total RNA from each group was extracted with TRIzol reagent (Thermo Fisher Scientific), and 1 μg of RNA was converted to cDNA using a QuantiTect Reverse Transcription Kit (Qiagen, Antwerpen, Belgium) and amplified using a power SYBR^®^ kit (Thermo Fisher Scientific) on an ABI 7500 Sequence Detection System. The qPCR thermal profile was 30 s of preincubation at 95 °C for one cycle, followed by 40 cycles of 95 °C for 5 s and 60 °C for 34 s. The fold changes in targeted gene amplification were determined by normalization to the housekeeping genes 18 s and B2M by the 2^−ΔΔCT^ method. Each experiment was evaluated by three PCRs. The amplification was performed in 96-well plates purchased from Lonza and Qiagen as described previously.

### 2.11. Gene Expression Array Data Analysis

The quality of RNA obtained from each AML and HD lymphocyte sample was assessed based on the RNA integrity using a Bioanalyser 2100 (Agilent Inc., Diegem, Belgium). Total RNA (100 ng) was converted to double-stranded cDNA in a reverse transcription reaction. The obtained cDNA was converted and amplified to antisense cRNA and labeled with biotin in an in vitro transcription reaction using an HT 3′ IVT Affymetrix Express Kit. All steps were carried out according to the manufacturer’s protocol (Affymetrix) [25]. A mixture of purified and fragmented biotinylated cRNA and hybridization controls (Affymetrix) was hybridized on Affymetrix GeneChip arrays followed by staining and washing. To assess the raw probe signal intensities, chips were scanned using a Gene Chip scanner 3000 (Affymetrix). The GeneChip™ Human Genome U133 Plus 2.0 Array (Affymetrix), which analyzes over 47,000 transcripts, was used in the study. The GC-Robust MultiChip Average (GCRMA) [26] was used as part of the GCRMA package at the Bioconductor site (http://www.bioconductor.org) to preprocess the raw data (CEL files).

### 2.12. Statistical Analysis

Comparisons between groups were carried out by either the paired *t*-test or ANOVA followed by *t*-tests with Bonferroni correction as required (GraphPad Software, Inc., La Jolla, CA, USA). *p* < 0.05 was considered to indicate a statistically significant difference.

## 3. Results

### 3.1. Apoptotic Pathways Induced Fragility of Aml T Lymphocytes

**Figure 1:**
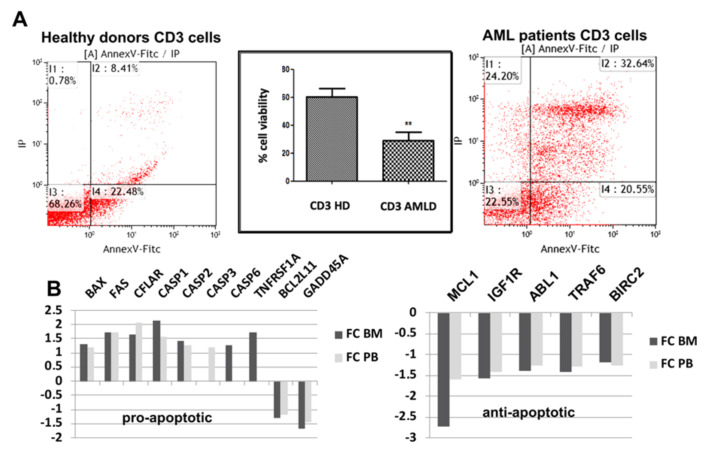
Fragility measurement of AML T lymphocytes. (**A**) The viability percentage of isolated and activated T lymphocytes (PHA/IL2) was measured in HD and untreated AML patients (=7) within six days post culture using an apoptosis test (cells were labeled with annexin-V-FITC and PI). The level of statistical significance was set at *p* < 0.01 (**). (**B**) Relative expression of proapoptotic and antiapoptotic genes in AML T lymphocytes (=27) compared to cells from HDs (=11) in both bone marrow (BM) and peripheral blood (PB) samples as determined using Affymetrix microarray (fold change >1.25 and *p* < 0.05).

AML is an acute cancer pathology manifested by a very high presence of leukemic blast cells in both the BM and the peripheral blood. The progression of AML is due in part to the inability of the immune response to eliminate leukemic cells. Several studies have shown the capacity of cancer cells to induce the apoptosis of certain immune cells, including T lymphocytes [27,28]. We have thus compared the cell death level between T lymphocytes isolated from AML patients and HDs by using annexin V-FITC and PI labeling (Figure 1A). After six days of activation with PHA and IL2, the T lymphocytes of AML patients had an increased death rate (75%) compared to that of cells from HDs (40%). We previously analyzed the expression profile of T cells isolated from both the BM and peripheral blood of AML patients using an Affymetrix microarray, and we compared it to that of lymphocytes from HDs (microarray data not shown). The GO term enrichment analysis of differentially expressed genes (DEGs) showed the association of more than 20 DEGs with the positive regulation of apoptosis (data not shown). Figure 1B shows the fold changes in upregulated apoptotic genes, including FAS, CFLAR, CASP1, CASP 2, CASP3, TNFRSF1A, and BAX, and downregulated antiapoptotic genes (MCL1, IGFR1, ABL1, TRAF6, and BIRC2) in AML patient BM and peripheral blood T lymphocytes compared to HD cells. These results show an increased fragility of T lymphocytes in patients with AML and suggest the involvement of apoptotic mechanisms in the induction of this fragility.

### 3.2. Myeloid Leukemia Cell Line-Derived Extracellular Vesicles Induce T Lymphocyte Cell Death

**Figure 2:**
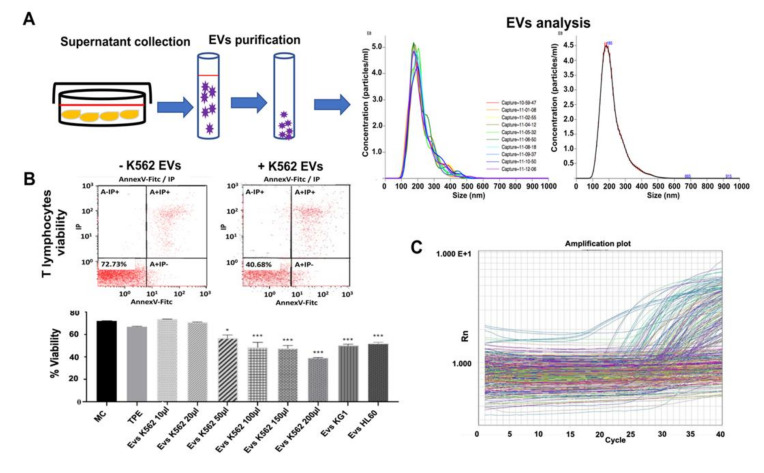
Myeloid leukemia cell line-derived EVs induce cell death in healthy donors (HD) T lymphocytes. (**A**) Characterization of K562-derived EVs. K562 EVs were characterized by nanoparticle tracking analysis (NTA). They ranged in size from 100 nm to 500 nm, with a mean value of 185 nm. The concentration of K562 EVs reached 28.05 × 10^9^ ± 25.05 × 10^7^ particles/mL. (**B**) The viability percentage of T lymphocytes (=3) was measured by an apoptosis test after the addition of K562 cell line-derived microvesicles (MVs) at different concentrations. The concentrations used for the MVs derived from HL60 and KG1 cells were similar to those used in the condition of 150 μl for the K562-MVs. MC: medium control. TPE: EV elution buffer. (**C**) Analysis of miRNA expression in the cargo of EVs purified from the supernatants of K562, KG1 and HL60 cells. The graph shows the expression curves of 385 miRNAs amplified using the TaqMan^®^ Array Human MicroRNA assay. The levels of statistical significance were set at *p* < 0.05 (*), *p* < 0.01 (**) and *p* < 0.001 (***).

To understand the mechanisms responsible for the fragility of AML T lymphocytes, we attempted to establish the link between leukemic EVs and the induction of T lymphocyte apoptosis. We proceeded first by purification and concentration of EVs from the supernatant of the leukemia myeloid line K562 (Figure 2A). The results showed that our EVs had an average size of 200 nm with a concentration of 28.5 × 10^9^ particles/mL. We then treated T cells from HDs with different concentrations of K562 EVs. As shown in Figure 2B, K562 EVs were capable of inducing T cell death depending on the concentration used. A total of 5.7 × 10^9^ particles decreased T cell viability by 30% compared to that of the control. We then analyzed the content of these EVs by TaqMan low-density array (TLDA). The results (Figure 2C and Table 2) showed the expression of several miRNAs with low Ct (Ct < 30), such as miR-19b, miR-21, miR-24, miR-20a, miR-17, and miR-222, indicating their abundance in K562-derived EVs. A similar effect was observed with EVs derived from the AML cell lines HL60 and KG1 (Figure 2A). Analysis of their cargo indicated that these miRNAs were also highly expressed (Table 2). Together, these results show that EVs derived from malignant leukemia cells can induce T lymphocyte cell death and that their cargo is rich in miRNAs, suggesting the role of certain miRNAs in the induction of the observed fragility of T lymphocytes in AML.

The pattern of miRNAs described in EV-K562 that are also expressed in both EVs of AML HL60 and KG1 cell lines. The numbers in the table represent the average of the cycle threshold (CT) values for the high-abundance miRNAs expressed in all three cell lines, CT < 30.

### 3.3. mir-21 Expression in the Aml Bone Marrow Tumor Microenvironment

**Figure 3:**
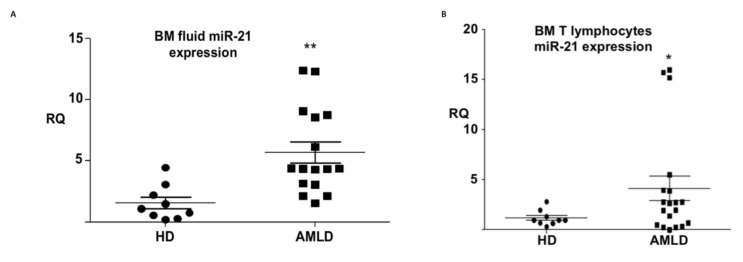
Relative expression level of miR-21 in bone marrow. (**A**) Bone marrow-circulating miR-21 expression in AML patients (*n* = 16) compared to HDs (*n* = 9). (**B**) MiR-21 is upregulated in AML patient T lymphocytes (*n* = 19) compared to HD lymphocytes (*n* = 9). Data obtained by quantitative RT-PCR amplification of miR-21 are plotted. All data were collected and statistically analyzed using Student’s *t*-test. Values are presented as the means of at least three independent experiments. The level of statistical significance was set at p < 0.01 (**) for BM-circulating AMLD vs HD and *p* ≤ 0.05 (*) for BM CD3 AML vs HD.

As shown in the previous section, miR-21 is abundantly expressed in EVs derived from various leukemia cell lines (KG1, HL60, and K562), and several studies have shown that miR-21 is overexpressed in the blasts of patients with AML as well as in myeloid leukemia cell lines (Supplementary Table S1). To date, the expression of miRNAs in the TME of the first location of AML initiation (namely, the BM) has not been analyzed. First, we analyzed the expression of miR-21 in the BM aspirations of patients with AML by reverse transcription-quantitative polymerase chain reaction (RT-qPCR). As shown in Figure 3A, circulating miR-21 was overexpressed (FC = 5.7; *p* < 0,01) in the BM bodily fluid of AML patients compared to HDs. We then proceeded to analyze the expression of this miRNA in T lymphocytes isolated from the BM of AML patients, as these cells are considered to be TILs in AML. Figure 3B shows upregulation (FC = 3.85; *p* ≤ 0.05) of miR-21 in AML TILs compared to lymphocytes from HDs. This overexpression of miR-21 in both malignant blasts and the TME (circulating miRNAs and T lymphocytes) suggests that this miRNA functions as both an oncogene and as a participant in tumor resistance through the vulnerability and dysfunction of TILs in AML.

### 3.4. mir-21 Induces T Lymphocyte Death Through Apoptotic Pathways

**Figure 4:**
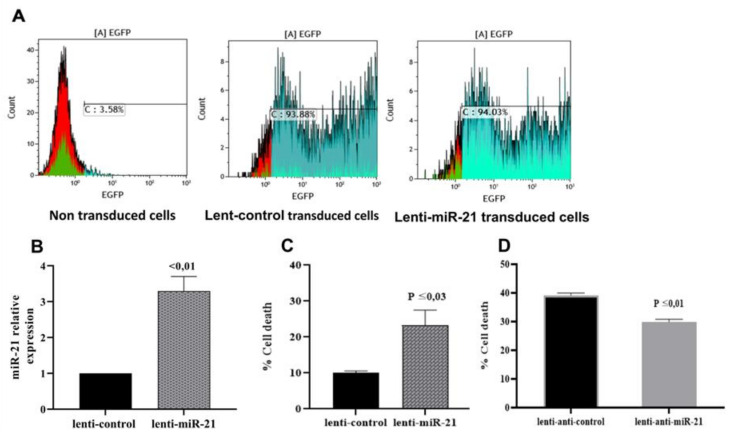
miR-21 induces cell death in T lymphocytes. (**A**) T cell lentivirus transduction efficiency: analysis using cytometry of the percentage of transduced human T lymphocytes (=3) by lentiviral vectors containing GFP and hsa-miR-21 or scramble as a control (=3). (**B**) Relative expression of miR-21 in lenti-miR-21-transduced cells (=3) compared to the control (=3). (**C**) MiR-21 induces HD T lymphocyte cell death. (**D**) Inhibition of miR-21 expression increases resistance to cell death in T lymphocytes (=3). All data were collected and statistically analyzed using Student’s *t*-test. Values are presented as the means of at least three independent experiments. The levels of statistical significance were set at *p* ≤ 0.03 and *p* < 0.01.

**Figure 5:**
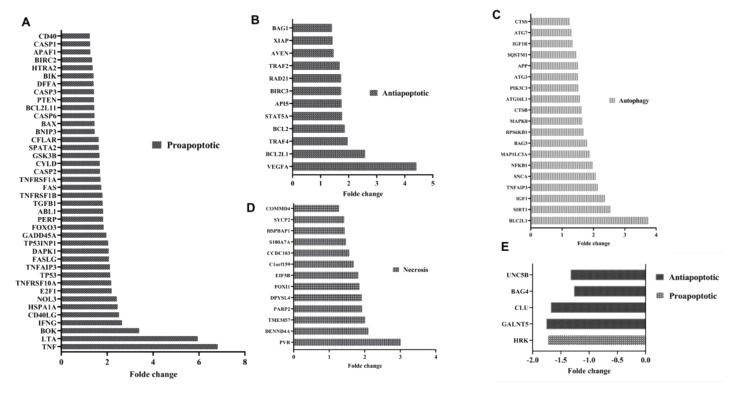
Gene expression analysis of cell death pathways induced by miR-21 (**A**–**E**). Analysis of the gene expression of cell death pathways in lenti-miR-21-transduced T lymphocytes (=3) compared to lenti-scramble-transduced T lymphocytes (=3). Relative expression of upregulated proapoptotic (**A**), antiapoptotic (**B**), autophagy (**C**), necrosis (**D**), and downregulated genes (**E**). All data were collected and statistically analyzed using Student’s *t*-test. The level of statistical significance was set at *p* < 0.05.

To establish the relationship between the fragility of AML T lymphocytes and their increased expression of miR-21, we transduced T lymphocytes isolated from HDs with lentivirus expressing miR-21 to mimic its effect in AML T lymphocytes. Transduction efficiency was monitored by flow cytometry and showed 94% GFP-positive cells (Figure 4A). Analysis of miR-21 expression in lenti-miR-21-transduced T lymphocytes showed increased expression compared to that in control cells (Figure 4B). Interestingly, the relative expression of miR-21 (FC = 3.45) in lenti-miR-21-transduced HD T lymphocytes compared to the lenti-control was very similar to that of AML T lymphocytes compared to HD cells (FC = 3.85) (Figure 4B). Cell viability analysis showed increased cell death in T lymphocytes transduced by lenti-miR-21 compared to that of the control (Figure 4C). Then, we proceeded to analyze the miR-21 inhibitory effect in weakened T lymphocytes using lenti-antagomiR-21. As shown in Figure 4D, miR-21 inhibition decreased the cell death rate of T lymphocytes. To confirm these results, we investigated the molecular pathways activated in miR-21-induced T cell death. We analyzed the expression of 170 genes involved in cell death and apoptosis (cell death pathway gene array from Qiagen and apoptosis pathway gene array from Lonza), revealing significant increases in the expression levels of different cell death pathway-related genes. Forty proapoptotic genes were upregulated, including CASP 1, CASP3, CFLAR, BAX, FAS, GADD45A, TP53, TNFRSF1A DENND4A, and TP53INP1, while antiapoptotic genes, including BAG3, CLU, GALNT5, and UNC5B, were downregulated (Figure 5A–E). Our results showed that miR-21-induced cell death is associated with the deregulation of genes implicated in other cell death pathways, namely, autophagy and necrosis, implicating these pathways in cell death induced by miR-21. Interestingly, some of the upregulated proapoptotic genes in miR-21-transduced T lymphocytes were also upregulated in AML T lymphocytes compared to HD cells, including BAX, CASP1, CASP3, FAS, CFLAR, and TNFRSF1A (Figure 1B). Taken together, these results show that miR-21 plays a crucial role in the induction of AML T cell fragility and suggest the involvement of apoptotic pathways in T cell death induced by miR-21.

### 3.5. mir-21 Expression Increases T Cell Immunosuppressive Phenotypic Markers

**Figure 6:**
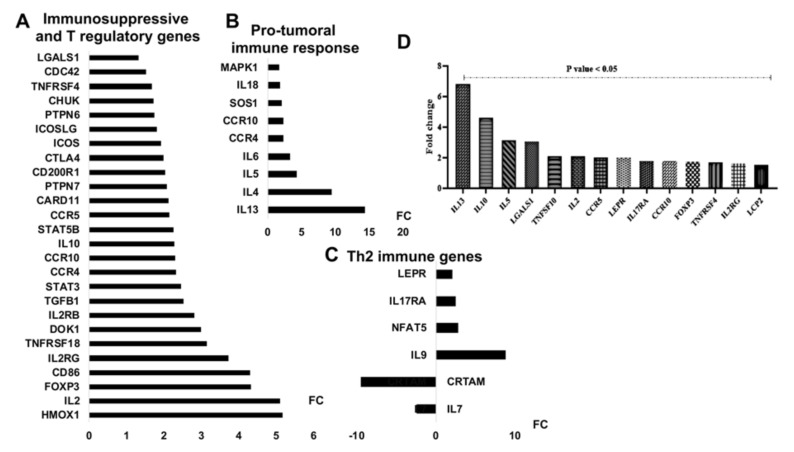
miR-21 induces a protumoral response in T lymphocytes. (**A**–**C**) Analysis of the expression of immune response genes in lenti-miR-21-transduced T lymphocytes (=3) compared to lenti-scramble-transduced T lymphocytes (=3). Relative expression of common genes related to T regulatory and immunosuppressive markers (**A**), Th2 immune genes (**B**) and genes related to the protumoral immune response (**C**). (**D**) Relative expression of genes related to the protumoral response in AML T lymphocytes (=27) compared to HD lymphocytes (=11). All data were collected and statistically analyzed using Student’s *t*-test. The level of statistical significance was set at *p* < 0.05.

To elucidate the role of miR-21 in regulating T lymphocyte function during AML, we evaluated the expression of genes implicated in the Treg and TCR signaling pathways after their transduction with lenti-miR-21. The results showed that miR-21 increased the expression of 26 genes considered to be Treg and immunosuppressive markers, including FOXP3, HMOX1, IL10, IL2RG, IL2RB, TGFB1, CCR10, CTLA4, ICOSLG, and LGALS1 (Figure 5A and Figure 6A). Ectopic expression of miR-21 also induced high levels of Th2 cytokines, including IL13, IL4, and IL5 (Figure 5B and Figure 6B), and cytokines implicated in Th9 and Th17 responses (Figure 5C and Figure 6C). These cytokines are specific to a suppressive immune response to cancer. Interestingly, miR-21 downregulated the IL7 and class I-restricted T cell-associated molecule (CRTAM) genes, which are known to be associated with the antitumoral response [29]. To confirm the link between the high expression of miR-21 in AML T lymphocytes and their inability to fight tumor cells, we analyzed the expression of protumoral cytokines using qRT-PCR-designed plates (as described previously). As shown in Figure 5D and Figure 6D, the freshly isolated AML T lymphocytes presented high levels (FC > 1,5; *p* < 0,05) of suppressive and protumoral cytokines, including IL13, IL10, FOXP3, IL2RG, IL2RB, LEPR, IL17RA, LGALS1, and HMOXA, compared to those in HD cells. Together, these results showed that miR-21 confers a suppressive and protumoral expression profile to HD T lymphocytes that largely resembles that of AML TILs.

## 4. Discussion

AML is a heterogeneous malignant disease that affects myeloid progenitors, resulting in the clonal proliferation of leukemic cells (blasts) in BM. In approximately 80% of patients, leukemic blasts quickly migrate to the PB. Leukemia, like many cancers, develops mechanisms to avoid immune destruction, and the interaction of blasts with BM and blood T lymphocytes contributes to both immune evasion and active immune suppression. TILs dysfunction is a major problem in not only the development of cancers but also their aggressiveness and resistance to therapeutic agents. In the case of AML, both cancer cells and cells in the TME (namely, mesenchymal stromal cells (MSCs), immune cells and stromal cells) play an important role in suppressing the effector function of T lymphocytes infiltrating the tumor focus (BM) [30,31]. However, in both human and mouse tumor models, these TILs appear to be capable of producing cytokines and being activated ex vivo [32,33] but are ineffective against cancer cells in vivo. In our work, we show that T lymphocytes freshly isolated from AML patients and cultured for one week are very fragile, presenting an increased cell death level in comparison to that of HD T lymphocytes. This fragility continues to manifest itself even in the absence of contact with blast cells, thus suggesting that it is the consequence of the apoptotic pathways already induced in vivo due to disease progression. The transcriptomic analysis and comparative profile of these freshly isolated lymphocytes (AML T lymphocytes vs HD T lymphocytes) before their culture confirmed our hypothesis and showed the differential expression of genes involved in the apoptotic pathways. Recent data have reinforced our observations and showed that AML blasts directly alter CD8+ T cell viability, expansion, cosignaling and senescence marker expression in vitro [34]. DEGs in AML CD8+ T cells have also been implicated in the apoptosis signaling pathways; these DEGS include upregulated CASP1 and FASLG, which are downregulated in complete remission compared to no remission CD8+ T cells. However, in this study, the CD8+ T cells isolated from patients after their manipulation (1-freezing of peripheral blood mononuclear cells (PBMCs), 2-thawing, 3-negative selection and sorting by FACS) experienced enormous apoptotic stress, which could have biased the results both in vitro and in the transcriptomic profile due to the activation of several apoptotic signaling pathways following thawing and FACS sorting. In our work, we attempted to avoid these problems by the selection and direct manipulation of AML lymphocytes freshly isolated from patients without freezing, thawing or sorting, demonstrating that the lymphocytes of AML patients freshly isolated from both blood and marrow are very fragile. Several recent studies have reported that T cell apoptosis represents a different tumor resistance mechanism [28]. These observations could explain, at least in part, the ineffectiveness of T lymphocytes in stopping the development of AML and must be considered in cellular immunotherapy when using CAR-T cells in AML. The fragility of patient lymphocytes could be an obstacle in increasing the efficiency of genetic engineering and the selection of these cells. In addition, transducing these cells may also not be very effective since they could remember the induction of apoptotic pathways despite their transduction. Like the use of inducible caspase 9 (iCasp9) as a suicide gene strategy to control the safety of CAR-T cells [35], we can imagine the induction of genes that are capable of protecting these cells from apoptotic mechanisms.

We then aimed to elucidate the endogenous or exogenous molecular mechanisms that could be responsible for the fragility of patient T lymphocytes. It is known that leukemic blasts play a critical role in modulating the T cell response, and T cell exposure to AML blasts in vitro leads to T cell apoptosis, inhibition of proliferation, and downregulation of costimulatory molecule expression. Monocytic leukemia cells produce ROS, which kill T cells and NK cells by triggering poly (ADP-ribose) polymerase-1–dependent (PARP-1–dependent) apoptosis [36]. This ability of tumor cells to induce cell death through the activation of T cell apoptosis, mainly by releasing microvesicles, has been observed in several types of cancers [37,38]. In our work, we showed that EVs derived from myeloid leukemia cell lines induce HD T lymphocyte cell death. Recent studies on exosomes derived from AML have shown their ability to suppress residual hematopoietic function and cause increased apoptosis in osteoprogenitor cells [39]. We then analyzed the cargo of myeloid leukemia cell EVs. A wide range of molecules are transported by EVs, including mRNAs, miRNAs, proteins, molecular chaperones, and signaling molecules. Our results showed a strong presence of oncogenic miRNAs in K562-, KG1-, and HL60-derived EVs that are known to be overexpressed in these malignant cell lines and in AML blasts (namely, miR-21, miR-222 and miR-24) [40,41,42,43,44,45]. Microvesicles derived from cancer cells play an important role in TME changes via the release of their content, including miRNAs, in target cells. Umezu et al. first reported that pre-miR-92a was transferred from leukemic K562 into the cytoplasm of endothelial cells [46]. To highlight the role of these miRNAs in the fragility of AML T lymphocytes, we herein focused on miR-21, which is known as an oncogene based on its role in AML and other cancers. In fact, miR-21 is overexpressed in AML blasts, and its inhibition in vitro induces the death of malignant myeloid cells [47]. In addition, in other cancers, including oral squamous cell carcinoma, exosomal miR-21 derived from tumor cells activates the downstream pathway PTEN-PD-L1 in myeloid-derived suppressor cells (MDSCs), which further improves immune tolerance in the TME [11]. We showed that circulating miRNA-21 is highly expressed in the BM aspirates of AML patients compared to those of HDs. This strong presence of circulating miRNA-21 in the BM could originate from blast cells, which express high levels of this miRNA, at diagnosis (Supplementary Table S1). Interestingly, we showed that in addition to its high level in BM aspirate, miR-21 is highly expressed in infiltrating T lymphocytes freshly isolated from the BM of AML patients compared to that of HDs. This result suggests that in addition to its exogenous role, miR-21 could play an endogenous role in the fragility of AML T cells. In fact, this hypothesis has been demonstrated in certain cancer models. Chia-Hsin Hsieh et al. showed that in human head and neck squamous cell carcinoma, cancer cell lines promote M2-like polarization of tumor-associated macrophages by delivering miR-21-abundant exosomes [48]. Furthermore, a study conducted by He et al. suggested that circulating miRNA-21 could have an impact on immune cells through Toll-like receptor signaling [49]. To confirm this role, we increased the expression of miR-21 in T lymphocytes of HDs by lentiviral transduction. We confirmed that the relative expression level of miR-21 after transduction of normal lymphocytes resembled that observed in T lymphocytes of patients, which allowed us to mimic in vitro its effect observed in vivo. Our results showed that the ectopic expression of miR-21 induced HD T lymphocyte cell death, while its inhibition significantly decreased the observed apoptosis. Interestingly, analysis of the transcriptomic profile of genes involved in the various cell death pathways showed a predominance of apoptotic pathways (more than 20 proapoptotic genes were upregulated), suggesting that miR-21 induces cell death in T lymphocytes through apoptosis involving the activation of caspases. The deregulation of genes involved in other cell death pathways (namely, autophagy and necrosis) following the overexpression of miR-21 suggests their possible involvement in cell death induced by miR-21. The interplay between different cell death pathways is known and is considered a complex process due to the multiple roles of certain genes, such as caspase 1. Kohsuke Tsuchiya et al. demonstrated that caspase-1 can initiate apoptosis involving the Bid-caspase-9-caspase-3 axis in the absence of gasdermin D (GSDME), which can elicit secondary necrosis/pyroptosis [50]. Interestingly, our transcriptomic analysis of AML TILs showed that a set of these genes (namely, ATG3, BAX, CASP1, CASP2, CASP3, CASP6, CFLAR, and FAS), was upregulated compared to that in HD lymphocytes, thus suggesting that their expression could be partly causing miR-21 overexpression in these cells. Together, these results showed that miR-21 induces cell death in T lymphocytes through different pathways, including the upregulation of apoptosis genes. Some of these genes were also upregulated in AML TILs in association with their high level of miR-21 expression. In addition to upregulated proapoptotic genes, ectopic miR-21 expression was associated with the decreased expression of some antiapoptotic genes, namely, UNC5B, BAG4, GALNT5, and clusterin (CLU). CLU was identified as a specific miRNA-21 gene target with functional significance [51], which suggests its direct implication in T cell death induced by miR-21. The fragility of TILs seems to be associated with a general dysfunction of their response in the tumor focus. In melanoma, Horton et al. found that TIL apoptosis was associated with antigen-induced T cell dysfunction, possibly due to the accumulation of DNA damage, and another group showed that miR-21 negatively impacts T lymphocyte function [52]. In previously published work, we showed that transduction of miR-21 into human non-Treg cells conferred a Treg cell phenotype with increasing FOXP3 levels [53]. The expression profile of regulatory T cell-specific genes and T cell receptor signaling pathway components in miR-21-transduced T cells revealed that up to 26 genes known to be associated with Tregs and immunosuppression in tumors were upregulated, including FOXP3, IL10, IL2RG, IL2RB, CTLA4, and LGALS1. MiR-21 also strongly induced the expression of Th2 and protumoral response markers, including IL13, IL4 and IL5, and downregulated antitumoral IL7 and CRTAM (FC = −9.3). The major histocompatibility complex (MHC) CRTAM is an Ig domain-containing and activation-induced surface receptor predominantly expressed on activated CD8+ T cells and NK/NKT cells. It promotes cytotoxicity and IFNγ secretion, favoring immunosurveillance [54]. Indeed, ectopic expression of CRTAM in T cells induced the production of IFN-γ, the expression of cytotoxic T lymphocyte-related genes, and cytotoxic activity [55]. Interestingly, CRTAM is cited in miRDB (online database for miRNA target prediction) as being targeted by miR-21, which is predicted to have potential target sites in its 3′-UTR at position 1163 (TAAGCTA) (http://mirdb.org/cgi-bin/target_detail.cgi?targetID=836188). Our results also showed the upregulation of protumoral Th9 and Th17 markers such as IL9 and IL17RA [56]. Together, these results show that miR-21 can confer an immunosuppressive and protumoral expression profile on T lymphocytes. Functional depletion of T cells and a high Treg cell level appear to be operational in AML disease [7]. Better understanding these phenomena and their underlying molecular mechanisms is essential for the rational integration of immunotherapeutic strategies for combatting AML. Our results also showed that a set of described protumoral immune genes, including IL10, FOXP3, IL13, IL5, IL2RG, IL2RB, LGALS1, LEPR, CCR10, and IL17RA, was upregulated in AML TILs compared to HD cells. This shows, on the one hand, that these genes are responsible for the immune escape of AML. On the other hand, the relative similarity between the protumoral immune profiles of AML TILs and miR-21-transduced HD T lymphocytes strongly suggests the direct involvement of miR-21 in AML TIL apoptosis and dysfunction.

Several clinical trials for suppressing miR-21 are currently being conducted. For example, different miRNA-targeted agents are being tested in patients such as miR-RX34 for liver cancer [57], ADM-21 for bladder cancer, and RG-012 in Alport syndrome [58]. In the light of the literature, miR-21 might be considered as a potential therapeutic target for several cancer types including AML [18,59,60]. To date, targeting miR-21 has shown promising results in mouse models of multiple myeloma [61], breast cancer and colon cancer [11]. Recently, an engineered exosome, designed by Liang et al., which could deliver chemotherapeutic drugs and miR-21 inhibitor oligonucleotide showed potent anti-cancer effect in colon cancer mouse models [62]. For AML, Chan Li et al. have shown that miR-21 promotes malignant cells proliferation in vivo and suggests it as a potential target for AML [18]. Interestingly, a recent study on a mouse model of lung cancer has shown that targeting this miR restores the antitumor response of macrophages and CD8+ resulting in a strong decrease in tumor burden [63,64]. This ability of targeting miR-21 to inhibit cancer cells proliferation and to induce their apoptosis (including AML), to reduce tumor burden in vivo and to restore the anti-tumor response pushes us to propose it as an immunotherapeutic target to fight against AML. In addition, RG-012, an inhibitor of miR-21 developed by Regulus Therapeutics (http://regulusrx.com/), is currently being investigated in the treatment of other malignant diseases. Possible ways of targeting miR-21 may include using EVs loaded with locked nucleic acid (LNA) antagomiR-21 in a murine AML model. Another approach could consist on the isolation of T lymphocytes from mice with AML, their transduction with antagomir-21 and finally their reinjection in this model. It is likely miR-21 is not the only cause of the aberrant response of AML TILs, as several miRNAs could participate in this phenomenon. Recently, our team observed that other miRNAs were upregulated in the BM TME and had a negative effect on T lymphocytes (data not shown). Thorough analysis of the AML TIL miRNA profile could help elucidate their molecular signature and the pathways involved to highlight new immunotherapeutic targets. Further information and basic research are required before miR-21 can be used extensively as an approved biomarker and therapeutic target, but its upregulation in a large number of cancers, as well as its relationship with many processes that regulate cancer progression, make it a promising molecule in cancer and in RNA-based therapies. Collectively, miR-21 is an appealing candidate because of all the carcinogenic processes it has been traced to. Targeting miR-21 could therefore serve as therapeutic strategy for therapy of AML.

## 5. Conclusions

This original study shows that T lymphocytes of patients with AML are very fragile due to the activation of different apoptotic pathways. We have observed that miR-21 is overexpressed in the TME of AML patients and may therefore be transferred to lymphocytes through EVs released from cancer cells. Finally, we demonstrated the ability of miR-21 to induce apoptosis in HD T lymphocytes and to confer them an immunosuppressive and protumoral expression profile, as observed in AML TILs. Increased understanding of how miRNAs mediate these roles may impact future AML immunotherapy. These results could help to better understand the mechanism orchestrating TILs immunosuppressive function to identify new targets for the treatment of AML.

## Figures and Tables

**Table 1:** Summary of acute myeloid leukemia (AML) patient clinical details used for the analysis.

Clinical details of AML patients (27)	Number
**Sex and age**	
Males	13
Females	14
Age < 55y	9
Age ≥ 55y	18
**Classification of AML patients**	
***Cytogenetic abnormalities***	
Normal Karyotype	3
Chromosome 9 deletion	2
Inversion (16) MYH11-CBFB	2
Translocation (t8;21)	2
Translocation (t15;17)	3
Translocation (X:21) (p11;q22)	1
trisomy 8	1
complex Karyotype	1
***WHO 2016 system***	
AML with recurrent genetic abnormalities (t8;21)	2
AML with recurrent genetic abnormalities (inv 16)	2
AML with recurrent genetic abnormalities (t15;17)	3
Acute monoblastic/monocytic leukemia	1
Pure erythroid leukemia	1
AML not otherwise specified (NOS)	15
unknown	3
***ELN 2017 genetic risk stratification***	
Favorable	10
Intermediate	3
Adverse	10
unknown	4

The classification of AML patients was performed according to WHO 2016 classification [1]. The genetic risk stratification of AML patients was done according to 2017 European LeukemiaNet genetic risk stratification [1]: Favorable = good prognosis; IT = intermediate prognosis; adverse = adverse risk.

**Table 2:** Common miRNAs expressed in extracellular vesicles (EVs) derived from myeloid leukemia cell lines.

EV-Derived miRNAs	K562 Mean Ct (SD)	KG1 Mean Ct (SD)	HL60 Mean Ct (SD)
hsa-miR-145	28.547 (1.034)	29.522 (1.064)	29.522 (1.064)
hsa-miR-484	27.792 (0.812)	28.481 (1.114)	28.481 (1.114)
hsa-miR-29a	28.463 (1.235)	27.747 (0.848)	27.747 (0.848)
hsa-miR-25	27.750 (1.350)	27.739 (0.707)	27.739 (0.707)
hsa-miR-425-5p	29.410 (1.167)	27.454 (1.213)	27.454 (1.213)
hsa-miR-186	27.534 (1.156)	26.930 (1.056)	26.930 (1.056)
hsa-miR-532	29.952 (1.000)	26.685 (0.619)	26.685 (0.619)
hsa-miR-210	26.659 (1.228)	26.656 (0.,904)	26.656 (0.904)
hsa-miR-27a	29.696 (1.184)	26.572 (0.798)	26.572 (0.798)
hsa-miR-320	25.836 (0.844)	26.540 (1.093)	26.540 (1.093)
hsa-miR-15b	28.530 (1.134)	25.739 (0.715)	25.739 (0.715)
hsa-miR-494	26.762 (1.276)	25.598 (1.387)	25.598 (1.387)
hsa-let-7^e^	29.536 (1.014)	25.354 (0.876)	25.354 (0.876)
hsa-miR-19a	29.703 (1.226)	24.629 (0.756)	24.629 (0.756)
hsa-miR-483-5p	26.502 (1.077)	24.592 (0.913)	24.592 (0.913)
hsa-miR-20b	28.791 (0.746)	23.807 (0.999)	23.807 (0.999)
hsa-miR-30c	24.754 (0.762)	23.778 (0.975)	23.778 (0.975)
hsa-miR-21	27.538 (1.215)	23.750 (1.082)	23.750 (1.082)
hsa-miR-16	26.564 (1.331)	23.693 (0.867)	23.693 (0.867)
hsa-miR-30b	26.688 (0.844)	23.656 (0.811)	23.656 (0.811)
hsa-miR-92a	24.688 (1.311)	23.539 (1.175)	23.539 (1.175)
hsa-miR-221	29.812 (1.008)	23.398 (1.106)	23.398 (1.106)
hsa-miR-146b	21.782 (1.058)	22.850 (1.152)	22.850 (1.152)
hsa-miR-142-3p	28.549 (1.094)	22.804 (0.799)	22.804 (0.799)
hsa-miR-191	22.488 (0.917)	22.626 (1.328)	22.626 (1.328)
hsa-miR-342-3p	24.720 (0.773)	22.442 (1.177)	22.442 (1.177)
hsa-miR-222	27.684 (0.892)	21.850 (1.156)	21.850 (1.156)
hsa-miR-24	26.898 (1.107)	21.815 (1.257)	21.815 (1.257)
hsa-miR-20a	24.698 (0.668)	21.463 (1.113)	21.463 (1.113)
hsa-miR-17	23.745 (1.036)	19.910 (1.037)	19.910 (1.037)
hsa-miR-106a	23.721 (0.986)	19.773 (0.746)	19.773 (0.746)
hsa-miR-223	20.705 (0.886)	18.706 (1.167)	18.706 (1.167)
hsa-miR-19b	23.768 (1.284)	18.478 (0.980)	18.478 (0.980)

## Supplementary Materials

The following are available online at https://www.mdpi.com/2073-4409/9/9/2053/s1, Table S1: miR-21 expression profile in the literature.
