# Supplementary material for: Impact of Bone Marrow miR-21 Expression on Acute Myeloid Leukemia T Lymphocyte Fragility and Dysfunction
Source: Cells. 2020 Sep 8;9(9):2053. doi: 10.3390/cells9092053 (PMC7563595; doi:10.3390/cells9092053)
Supplement: Supplementary file 1 [file cells-09-02053-s001.pdf]

Supplementary data

Table S1: miR-21 expression profile in the literature

miR-21 upregulated in AML blasts	Cohort	Blast origin	References
AML blasts vs HD CD34+ cells	215 patients	Bone marrow	[63]
AML blasts vs HDCD34+ cells	27 patients	Bone marrow	[64]
NPM1 mutated vs NPM1 nonmutated	13 mutated vs 7 nonmutated patients	Bone marrow	[64]
AML PBMCs vs HD PBMCs	26 patients	(PB)	[65]
AML SKM-1 and HL-60 cells compared to normal bone marrow stromal HS-5 cells	2 myeloid leukemia cell lines vs normal BM stromal cells	HL60: PB<br>SKM-1: PB	[18]
AML cell lines vs HD PBMCs	5 myeloid leukemia cell lines vs normal BM stromal cells	HL60: PB<br>THP1: PB<br>KG1: BM<br>MEG-01: BM<br>K562: blood	[65]

Data from published results showing the expression of miR-21 in AML blasts from patients and myeloid leukemia cell lines compared to PBMCs or CD34+ cells from healthy donors or between different groups of patients related to their genetic risk. BM: bone marrow, PB: peripheral blood.
